# Urinary Catheter Causing Paracentesis-induced Circulatory Dysfunction

**DOI:** 10.5811/cpcem.24869

**Published:** 2025-01-01

**Authors:** José Guilherme Assis, Joana Rua

**Affiliations:** Centro Hospitalar de Trás-os-Montes e Alto Douro, Department of Medicine, Vila Real, Portugal

**Keywords:** urinary bladder diseases, paracentesis, shock

## Abstract

**Case Presentation:**

A 78-year-old male was admitted to the hospital due to acute-on-chronic liver failure with spontaneous bacterial peritonitis. About six liters of a yellow, turbid fluid were collected via indwelling urinary catheter (UC) overnight. He subsequently developed neurological and cardiac dysfunctions. Imaging confirmed bladder perforation and intraperitoneal placement of the UC, establishing the diagnosis of paracentesis-induced circulatory dysfunction due to unintended ascitic fluid drainage. He was stabilized with albumin replacement. The UC was removed, and the bladder injury resolved spontaneously.

**Discussion:**

This case depicts a rare complication of urinary catheterization, which underscores the need for careful monitoring and prompt intervention to effectively manage unexpected catheter-related issues.

## CASE PRESENTATION

**Image 1:**
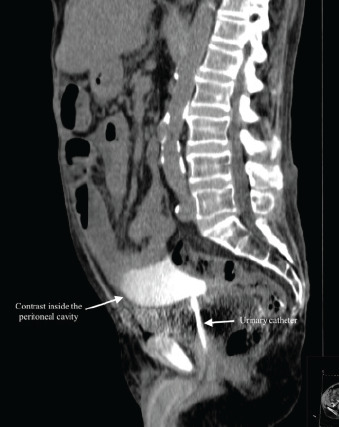
Abdominopelvic computer tomography (sagittal plane) displaying extravasation of contrast administered through the urinary catheter into the peritoneal cavity.

**Image 2:**
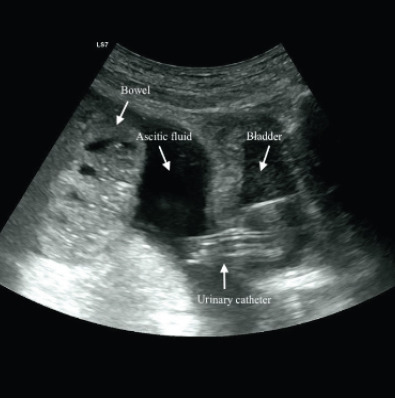
Abdominal ultrasonography confirming extrusion of the urinary catheter through the superior surface of the bladder associated with traumatic rupture.

A 78-year-old male with alcoholic cirrhosis and chronic renal disease with a longstanding indwelling urinary catheter (UC), previously placed because of obstructive uropathy, was admitted to the hospital due to acute-on-chronic liver failure associated with spontaneous bacterial peritonitis. On the fourth day of hospitalization approximately six liters of a yellow, turbid fluid, consistent with infected ascitic fluid, were unexpectedly drained via the UC through the night. This incident resulted in the patient developing somnolence and mild abdominal discomfort. A physical examination revealed pallor, hypotension (blood pressure of 53/29 millimeters of mercury), and sinus tachycardia (heart rate of 105 beats per minute), suggesting significant hemodynamic instability. Besides neurological and cardiac dysfunctions, acute kidney failure was also documented. The imaging studies, including abdominal ultrasonography and computed tomography, revealed extravasation of contrast material injected through the UC into the peritoneal cavity, indicating bladder perforation and confirming the intraperitoneal placement of the catheter (Images 1 and 2).

## DISCUSSION

Urinary catheterization is an essential procedure for the practicing healthcare professional.^1^ The most common complications include bacterial infection (occurring at a frequency of 1 per 100 to 1 per 1000 catheter days in patients with long-term UC), mechanical trauma (bladder perforation, urethral damage, and urinary leakage, typically affecting one-third of chronic patients) and, rarely, catheter toxicity.^2^,^3^ We report a unique case of circulatory dysfunction precipitated by inadvertent, large-volume drainage of ascitic fluid through a bladder perforation caused by a UC. To our knowledge, no prior cases of this specific complication have been described in the literature. Similar reports involve bladder rupture leading to urinary ascites, although these events are still infrequent.^4^ This unusual complication underscores the importance of careful monitoring, prompt diagnosis, and appropriate therapeutic interventions to manage catheter-related issues,^5^ especially in patients with complex medical histories. In this case, early intervention and appropriate management led to a positive outcome even in the face of severe complications.

**CPC-EM Capsule**

What do we already know about this clinical entity?

*Urinary catheters can cause complications such as infection, mechanical trauma (including bladder perforation), and catheter toxicity*.

What is the major impact of the image(s)?

*The images confirm bladder perforation and fluid extravasation through the urinary catheter, which is critical for diagnosing the cause of the unexpected ascitic drainage*.

How might this improve emergency medicine practice?

*Early recognition of catheter-related complications, timely use of imaging for diagnosis, and prompt treatment are essential to prevent severe outcomes*.
